# From aesthetic engagement to pro-environmental intention: the serial mediating roles of awe and green mindfulness in university students’ responses to traditional Chinese nature poetry

**DOI:** 10.3389/fpsyg.2026.1975372

**Published:** 2026-10-28

**Authors:** Hua Long, Jiali Shi, Quan Zhang, Dun Zhao, Xiaoxi Ma

**Affiliations:** 1 College of Agronomy and Biotechnology, Yunnan Agricultural University, Kunming, China; 2 College of Economics and Management, Yunnan Agricultural University, Kunming, China; 3 Office of Asset and Laboratory Management, Yunnan Agricultural University, Kunming, China

**Keywords:** aesthetic appeal, awe, green mindfulness, pro-environmental behavioral intention, sustainability education, traditional Chinese nature poetry

## Abstract

**Introduction:**

Universities provide an important setting for integrating cultural learning with sustainability education. This study examines the association between the perceived aesthetic appeal of traditional Chinese nature poetry and university students’ pro-environmental behavioral intention (PEBI), as well as the potential indirect associations involving awe and green mindfulness. Traditional Chinese nature poetry provides a culturally embedded aesthetic and educational context for examining these psychological relationships.

**Methods:**

Cross-sectional survey data were collected from 409 Chinese university students across four academic semesters and analyzed using partial least squares structural equation modeling (PLS-SEM) and artificial neural network (ANN) analysis.

**Results:**

Aesthetic appeal was positively associated with PEBI, awe, and green mindfulness. Significant indirect associations between aesthetic appeal and PEBI were observed through both awe and green mindfulness, together with a significant serial indirect association through these two variables. The model explained 43.7% of the variance in PEBI. ANN analysis further indicated that aesthetic appeal had the greatest relative predictive importance for PEBI, followed by green mindfulness and awe.

**Discussion:**

The findings suggest that self-transcendent emotion and environmentally oriented awareness may help explain the association between perceived aesthetic appeal and pro-environmental behavioral intention. These relationships should be interpreted as theoretically informed statistical associations rather than evidence of an established temporal or causal psychological process. By examining aesthetic evaluation, emotional experience, and environmental awareness within a culturally embedded educational context, this study contributes to understanding psychological correlates of pro-environmental intention and highlights potential connections between traditional Chinese poetry appreciation and sustainability education.

## 1 Introduction

Climate change is reshaping ecosystem stability and biodiversity while placing growing pressure on human health through extreme heat, food insecurity, and an increasing burden of disease. These interconnected risks continue to challenge global sustainability (IPCC et al., 2023; Pörtner et al., 2023; Romanello et al., 2024). Technological innovation, policy support, and institutional governance remain essential, but these measures alone are unlikely to be sufficient without public willingness to adopt environmentally responsible practices. This issue is particularly relevant to younger generations, whose environmental awareness and everyday choices are closely connected to future sustainability trajectories (Shafiei and Maleksaeidi, 2020; Shutaleva et al., 2022). Higher education therefore has a role that goes beyond simply transmitting knowledge: universities provide important settings in which students may develop the values and practical capacities relevant to environmental challenges through formal curricula, campus initiatives, and sustainability education (Hammer and Lewis, 2023). Evidence from Chinese universities likewise suggests that environmental education, green campus practices, and institutional support are associated with stronger green intentions and pro-environmental behaviors (Chen et al., 2025). Against this background, strengthening university students’ pro-environmental behavioral intentions has become an increasingly relevant goal of sustainability-oriented higher education.

Pro-environmental behavioral intention (PEBI) refers to an individual’s willingness to engage in environmentally responsible actions and is commonly regarded as a proximal antecedent of actual behavior (Zhang et al., 2026). Among university students, PEBI has been linked to environmental beliefs, attitudes, and intrinsic motivation, particularly in the context of climate-related action (Juma-Michilena et al., 2023). On campus and in everyday life, such engagement may take the form of conserving energy, recycling, choosing greener products, reducing unnecessary resource use, or participating in environmental initiatives (Shafiei and Maleksaeidi, 2020; Takshe et al., 2023; Chen and Erfanian, 2026). A range of factors have been associated with these intentions and behaviors, including environmental knowledge (Wang and Zhang, 2021), connectedness to nature (Guazzini et al., 2025), biospheric values, environmental attitudes, and social norms (Chen and Erfanian, 2026). Yet greater environmental knowledge is not necessarily accompanied by correspondingly stronger pro-environmental action. Singh et al. (2026), for example, identified a persistent “knowledge–action gap” among university students. This gap suggests that informational factors alone cannot fully account for differences in PEBI. Emotional responses, cognitive orientations, and culturally situated experiences may also be associated with such differences.

Two psychological processes that may be particularly relevant to understanding the relationship between nature-related aesthetic appreciation and pro-environmental behavioral intention are awe and green mindfulness. Awe is a self-transcendent emotion that often arises when individuals encounter experiences perceived as vast and requiring a shift in their existing mental frameworks. Natural environments represent one of the most frequently studied contexts in which awe is experienced, and previous studies have suggested that awe-inspiring experiences in nature may be associated with stronger pro-environmental responses (Chirico et al., 2023; Liu et al., 2024).

Green mindfulness represents an environmentally specific form of mindful awareness, referring to individuals’ attentive and reflective awareness of environmental conditions and the ecological consequences of their actions. Unlike general trait mindfulness, which reflects a broader tendency to attend to present-moment experiences with openness and awareness, green mindfulness specifically emphasizes environmentally oriented cognition and reflection on human–nature relationships. Recent research indicates that green mindfulness may be associated with pro-environmental behaviors and intentions, although the strength and nature of this association may vary depending on the specific dimension of mindfulness examined (Bontempo et al., 2026a; Karl and Stanley, 2024). Among university students, environmental knowledge, environmental awareness, and perceived university environmental responsibility have been linked to higher levels of green mindfulness (Rathakrishnan et al., 2025), while green mindfulness has also been associated with specific behaviors such as reducing single-use plastic consumption (Tanhayi et al., 2026).

Taken together, awe and green mindfulness represent complementary psychological processes. Awe reflects an affective response involving self-transcendence and an expanded orientation toward the natural world, whereas green mindfulness reflects attentive and reflective awareness of environmental information and behavioral consequences. These two processes may therefore provide complementary perspectives for understanding variations in pro-environmental behavioral intention in relation to nature-related aesthetic appreciation.

Culturally embedded experiences may shape how individuals perceive, interpret, and assign meaning to nature. Scholarship in the environmental humanities has suggested that poetry can do more than represent environmental change; through imagery and emotion, it may also provide a context for reflecting on ecological responsibility and relationships with the natural world (Xie, 2023). Traditional Chinese poetry frequently incorporates natural imagery, including mountains, rivers, vegetation, animals, and seasonal changes, with such imagery often intertwined with human emotions and cultural meanings. Its theoretical relevance lies not simply in the presence of natural themes, but in the integration of natural landscapes, emotional experience, and culturally grounded understandings of human–nature relationships within a distinctive aesthetic form.

Importantly, the aesthetic qualities of these poems extend beyond sensory pleasure or formal beauty. Many nature poems portray expansive landscapes, enduring natural processes, and broader relationships between humans and the natural world, features that may evoke perceptions of vastness, transcendence, and self-expansion commonly associated with awe. Previous empirical research on classical Chinese poetry has suggested that poetic imagery is associated with variations in emotional experience and aesthetic responses (Qin et al., 2025). In the present study, perceived aesthetic appeal refers to students’ subjective evaluations of the beauty, attractiveness, and overall appeal of traditional Chinese nature poetry. It reflects their appreciation of the aesthetic qualities of the selected poetic works rather than their literary knowledge or general aesthetic competence. Although these evaluations occur within a culturally meaningful context, aesthetic appeal is distinguished conceptually from the emotional experience of awe and environmentally oriented mindful awareness.

Accordingly, traditional Chinese nature poetry is not treated as merely another form of nature exposure. Rather, it provides a culturally embedded aesthetic and educational context in which natural landscapes, emotional expression, and culturally informed understandings of human–nature relationships are jointly represented. Examining students’ psychological responses within this context may offer insights into how aesthetic evaluation, emotional experience, and environmental awareness are related. Importantly, the poetry materials serve as a common context for the participants rather than an independently manipulated stimulus, and the present study does not seek to establish the causal effects of poetry exposure.

Despite these theoretical connections, relatively little is known about how university students’ perceived aesthetic appeal of traditional Chinese nature poetry is associated with their pro-environmental behavioral intention, particularly when awe and green mindfulness are considered together. Existing research provides a basis for examining associations between aesthetic appreciation, self-transcendent emotion, environmentally oriented awareness, and behavioral intention. However, the relationships among these constructs within the specific context of traditional Chinese nature poetry appreciation remain insufficiently understood. In particular, the potential indirect associations involving awe and green mindfulness, both separately and sequentially, warrant further investigation.

To address this gap, the present study adopts an association-based conceptual framework to examine the relationships among perceived aesthetic appeal, awe, green mindfulness, and PEBI. Perceived aesthetic appeal represents students’ subjective evaluation of the poetry’s aesthetic qualities, while awe and green mindfulness represent distinct affective and environmentally oriented attentional constructs, respectively. PEBI reflects the behavioral intention outcome of interest. Using cross-sectional data from undergraduate students at Yunnan Agricultural University in China, the study investigates the direct associations among these variables and the theoretically specified indirect associations involving awe and green mindfulness. The hypothesized relationships are examined as statistical associations rather than as evidence of an established temporal or causal psychological sequence.

By doing so, the study contributes to research on pro-environmental behavioral intention in several ways. First, it examines aesthetic evaluation in the culturally specific context of traditional Chinese nature poetry appreciation, extending the range of educational settings considered in research on environmental intentions. Second, it considers self-transcendent emotion and environmentally oriented mindful awareness within a common analytical model, offering a more integrated understanding of their statistical relationships with aesthetic appeal and PEBI. Third, the findings provide a basis for further investigation of potential connections among traditional cultural education, aesthetic education, and sustainability education in Chinese higher education. These contributions concern psychological associations within a specific educational context rather than the demonstrated effectiveness of poetry-based interventions.

## 2 Theoretical background and hypothesis development

### 2.1 Conceptual framework

The present study adopts an association-based conceptual framework to examine the relationships among perceived aesthetic appeal, awe, green mindfulness, and pro-environmental behavioral intention (PEBI) within the context of traditional Chinese nature poetry appreciation. Drawing on research on aesthetic appreciation (Leder et al., 2004), awe (Keltner and Haidt, 2003), and green mindfulness (Chen et al., 2014), the framework integrates aesthetic evaluation, emotional experience, and environmentally oriented awareness to investigate their associations with behavioral intention.

Perceived aesthetic appeal represents students’ subjective evaluation of the beauty, attractiveness, and overall appeal of traditional Chinese nature poetry. Awe refers to a self-transcendent emotional experience characterized by perceived vastness and cognitive accommodation, whereas green mindfulness reflects attentive awareness of environmental information, human–nature relationships, and the ecological consequences of personal actions. PEBI represents students’ intentions to engage in environmentally responsible behaviors. These constructs are conceptually distinct but may be theoretically related within the shared cultural and educational context of poetry appreciation.

Based on these theoretical considerations, the proposed model examines the direct associations among aesthetic appeal, awe, green mindfulness, and PEBI, as well as the indirect associations involving awe and green mindfulness, both independently and sequentially. Traditional Chinese nature poetry provides the common research context rather than an independently manipulated stimulus. Accordingly, the hypothesized relationships represent theoretically informed statistical associations rather than established causal mechanisms or temporal psychological processes.

### 2.2 Aesthetic appeal and pro-environmental behavioral intention

Aesthetic appeal refers to an individual’s subjective evaluation of the aesthetic qualities of an object or experience, including perceptions of beauty, attractiveness, and personal appreciation (Gao and Guo, 2018; Wang et al., 2021). In the context of traditional Chinese nature poetry, perceived aesthetic appeal specifically reflects students’ evaluations of the beauty, attractiveness, and overall appeal of the selected poems. It therefore represents a context-specific aesthetic judgment rather than general aesthetic competence, literary knowledge, or a direct measure of environmental awareness.

Aesthetic appreciation involves more than passive perception. Cognitive models of aesthetic experience suggest that aesthetic judgments are associated with perceptual analysis, information integration, and evaluative processing (Leder et al., 2004). Traditional Chinese nature poetry provides a distinctive context for such evaluations because natural imagery contributes substantially to its aesthetic expression. Mountains, rivers, vegetation, birds, and seasonal changes are frequently integrated into poetic language and atmosphere, making representations of nature an important component of the aesthetic object (Cai, 2007; Li and Ryan, 2017). Consequently, positive aesthetic evaluations of these poems may be associated with appreciation of the natural landscapes and imagery they portray, although aesthetic appeal itself does not directly measure ecological values or environmental concern.

Previous research provides a theoretical basis for examining the relationship between aesthetic appreciation and pro-environmental outcomes. Diessner et al. (2018) reported a positive relationship between valuing the beauty of nature and pro-environmental behavior, while Diessner and Niemiec (2023) found that appreciation of natural beauty was associated with pro-environmental behavior and behavioral intention. Although appreciation of natural environments differs from appreciation of their poetic representations, these findings suggest that aesthetic evaluation may be relevant to environmentally oriented intentions. In the present cultural context, students who report greater aesthetic appeal toward traditional Chinese nature poetry may also report stronger willingness to engage in environmentally responsible behaviors. Accordingly, the following hypothesis is proposed:

*H1*: The perceived aesthetic appeal of traditional Chinese nature poetry is positively associated with university students’ pro-environmental behavioral intention.

### 2.3 The indirect association involving awe

Awe is a self-transcendent emotion commonly associated with perceived vastness and a need for cognitive accommodation (Keltner and Haidt, 2003). Although aesthetic appreciation and awe may occur in related experiential contexts, they represent conceptually distinct constructs. Aesthetic appeal primarily concerns the subjective evaluation of beauty and attractiveness, whereas awe involves an emotional response to experiences perceived as exceeding ordinary frames of reference. Natural environments are frequently associated with awe because expansive landscapes and natural phenomena can convey vastness, grandeur, and a broader perspective on the individual’s place in the world (Shiota et al., 2007).

Traditional Chinese nature poetry provides a culturally meaningful context in which aesthetic evaluation and awe may be related. Representations of mountains, rivers, the sky, the moon, and seasonal change often convey expansive natural settings and relationships between humans and the wider natural world. Students who evaluate these poetic representations more positively may also report stronger emotional engagement with their imagery and atmosphere. Although such an association does not demonstrate that aesthetic appreciation causes awe, the conceptual connection between poetic representations of vastness and awe provides a basis for examining their relationship.

Awe may also be associated with pro-environmental behavioral intention because self-transcendent experiences have been linked to perspectives extending beyond immediate personal concerns. Piff et al. (2015) identified the diminished sense of self, commonly described as the “small self,” as an important characteristic of awe. Such experiences have been associated with reduced self-focus and greater attention to entities and concerns beyond the individual. Yang et al. (2018) found that awe was related to ecological behavior through connectedness to nature, while Monroy and Keltner (2023) discussed broader psychological relationships involving awe, reduced self-focus, and connectedness.

These findings suggest that individuals reporting stronger awe may also express greater concern for the natural environment and stronger intentions to engage in environmentally responsible actions. Within the present study, awe is therefore considered a potentially relevant emotional correlate of both perceived aesthetic appeal and PEBI.

Taken together, the literature provides a theoretical basis for expecting positive associations between aesthetic appeal and awe and between awe and PEBI. These relationships also support examining whether awe accounts for part of the statistical association between aesthetic appeal and PEBI. However, because the present research uses cross-sectional data, such an indirect association cannot establish that aesthetic evaluation temporally precedes awe or that awe causally transmits an effect on behavioral intention. Accordingly, the following hypotheses are proposed:

*H2*: University students’ perceived aesthetic appeal of traditional Chinese nature poetry is positively associated with their reported experience of awe during poetry appreciation.

*H3*: Awe is positively associated with university students’ pro-environmental behavioral intention.

*H4*: Perceived aesthetic appeal is expected to show a positive indirect association with pro-environmental behavioral intention through awe.

### 2.4 The indirect association involving green mindfulness

Green mindfulness refers to an environmentally oriented form of mindful awareness characterized by attention to environmental information, ecological concerns, and the potential consequences of human actions (Chen et al., 2014). Unlike general mindfulness, which concerns broader patterns of present-moment awareness, green mindfulness specifically emphasizes environmentally relevant information and reflection on human–nature relationships. Previous research has linked environmental cognition, awareness, and perceived environmental responsibility to green mindfulness (Chen et al., 2015; Rathakrishnan et al., 2025).

Aesthetic evaluation may be relevant to environmentally oriented awareness because aesthetic qualities can be associated with the allocation of attention and cognitive processing. Reppa and McDougall (2022) demonstrated that aesthetic appeal can influence visual search performance, although their findings do not imply that aesthetically attractive information automatically captures attention or establish the same relationship in poetry appreciation. Nevertheless, their research provides a broader theoretical rationale for considering possible relationships between aesthetic evaluation and attention.

In traditional Chinese nature poetry, natural imagery constitutes an important element of the aesthetic content. Landscapes, vegetation, animals, and seasonal changes are embedded in poetic expression and often accompanied by representations of human–nature relationships. Students who report stronger aesthetic appreciation may also be more attentive to these nature-related elements. Such attentiveness is conceptually relevant to green mindfulness, which concerns conscious awareness of environmental information and ecological consequences. However, because the present study does not directly measure changes in attention during poetry appreciation, this possibility is treated as a theoretically informed explanation rather than an established mechanism.

Direct empirical evidence concerning the relationship between perceived aesthetic appeal and green mindfulness remains limited. Nevertheless, research on aesthetic processing and environmentally oriented awareness provides a basis for examining whether these constructs are positively associated within a shared poetry appreciation context. Students who evaluate traditional Chinese nature poetry more positively may also report higher levels of green mindfulness, without implying that the poetry experience necessarily produced an increase in environmental awareness.

Green mindfulness may also be relevant to PEBI because environmentally oriented awareness is associated with attention to the ecological implications of everyday decisions. Barbaro and Pickett (2016) reported a relationship between mindfulness and pro-environmental behavior involving connectedness to nature. Apaolaza et al. (2022) further examined associations between mindfulness and pro-environmental behavior involving cognitive reappraisal and climate-change awareness. More specifically, Bernadip et al. (2026) identified green mindfulness as a positive predictor of pro-environmental behavioral intention among university students.

Together, these findings suggest that individuals reporting greater green mindfulness may also report stronger intentions to conserve resources, reduce waste, and engage in environmentally responsible practices. Accordingly, green mindfulness may help account statistically for part of the association between perceived aesthetic appeal and PEBI. Such an indirect association, however, should not be interpreted as evidence that aesthetic appreciation causes changes in green mindfulness or that these changes subsequently produce behavioral intentions. Based on these considerations, the following hypotheses are proposed:

*H5*: University students’ perceived aesthetic appeal of traditional Chinese nature poetry is positively associated with green mindfulness.

*H6*: Green mindfulness is positively associated with university students’ pro-environmental behavioral intention.

*H7*: Perceived aesthetic appeal is expected to show a positive indirect association with pro-environmental behavioral intention through green mindfulness.

### 2.5 The serial indirect association involving awe and green mindfulness

Although awe and green mindfulness represent distinct psychological constructs, existing theoretical perspectives suggest that they may be related. Awe involves self-transcendent emotional experience and has been associated with reduced self-focused attention and stronger perceptions of connectedness to the broader natural world (Shiota et al., 2007; Piff et al., 2015; Bai et al., 2017). Green mindfulness, by contrast, concerns attentive awareness of environmental information, human–nature relationships, and the consequences of human actions (Chen et al., 2014). Their conceptual distinction provides a basis for investigating whether affective experience and environmentally oriented awareness are statistically connected.

One possible explanation for this relationship concerns the attentional characteristics associated with self-transcendent emotions. Experiences of awe may be accompanied by a broader perspective extending beyond immediate personal concerns, potentially making wider environmental relationships more salient. This possibility is broadly consistent with the broaden-and-build perspective, which proposes that positive emotional experiences can broaden the scope of attention and cognitive processing (Fredrickson, 1998, 2001; Fredrickson and Branigan, 2005). However, awe should not be equated with positive emotion in general, and this theoretical perspective does not directly establish a causal relationship between awe and green mindfulness.

In the context of traditional Chinese nature poetry, representations of expansive landscapes, natural continuity, and human–nature relationships may provide meaningful content for both awe-related experiences and environmentally oriented awareness. Students reporting stronger awe during poetry appreciation may also report greater attention to environmental information and reflection on human–nature relationships. Previous findings linking awe to connectedness to nature and ecological behavior are compatible with this interpretation (Yang et al., 2018), although they do not directly demonstrate that awe precedes or produces green mindfulness.

The relationships among aesthetic appeal, awe, green mindfulness, and PEBI can therefore be examined within a theoretically specified serial indirect model. Perceived aesthetic appeal may be positively associated with awe, which may, in turn, be positively associated with green mindfulness. Green mindfulness may also be associated with stronger pro-environmental behavioral intention, as suggested by previous research on environmentally oriented awareness and behavioral intentions (Apaolaza et al., 2022; Bernadip et al., 2026).

Importantly, the proposed ordering reflects a theoretically plausible specification rather than an empirically established temporal process. Because all constructs were measured within a cross-sectional design, the analysis cannot determine whether awe occurred before green mindfulness or exclude alternative explanations for their observed relationships. The serial indirect association is therefore interpreted as a statistical pattern consistent with the proposed theoretical relationships, not as evidence of a confirmed causal chain. Accordingly, the following hypotheses are proposed:

*H8*: Awe reported during traditional Chinese nature poetry appreciation is positively associated with university students’ green mindfulness.

*H9*: Perceived aesthetic appeal is expected to show a positive serial indirect association with pro-environmental behavioral intention through awe and green mindfulness.

Figure 1 presents the conceptual model proposed in this study. Perceived aesthetic appeal represents students’ subjective evaluation of the aesthetic qualities of traditional Chinese nature poetry. Awe and green mindfulness represent distinct but potentially related emotional and environmentally oriented attentional constructs, respectively, while pro-environmental behavioral intention (PEBI) represents the outcome of interest. The model examines direct associations among these constructs and indirect associations involving awe and green mindfulness, both separately and sequentially.

**Figure 1:**
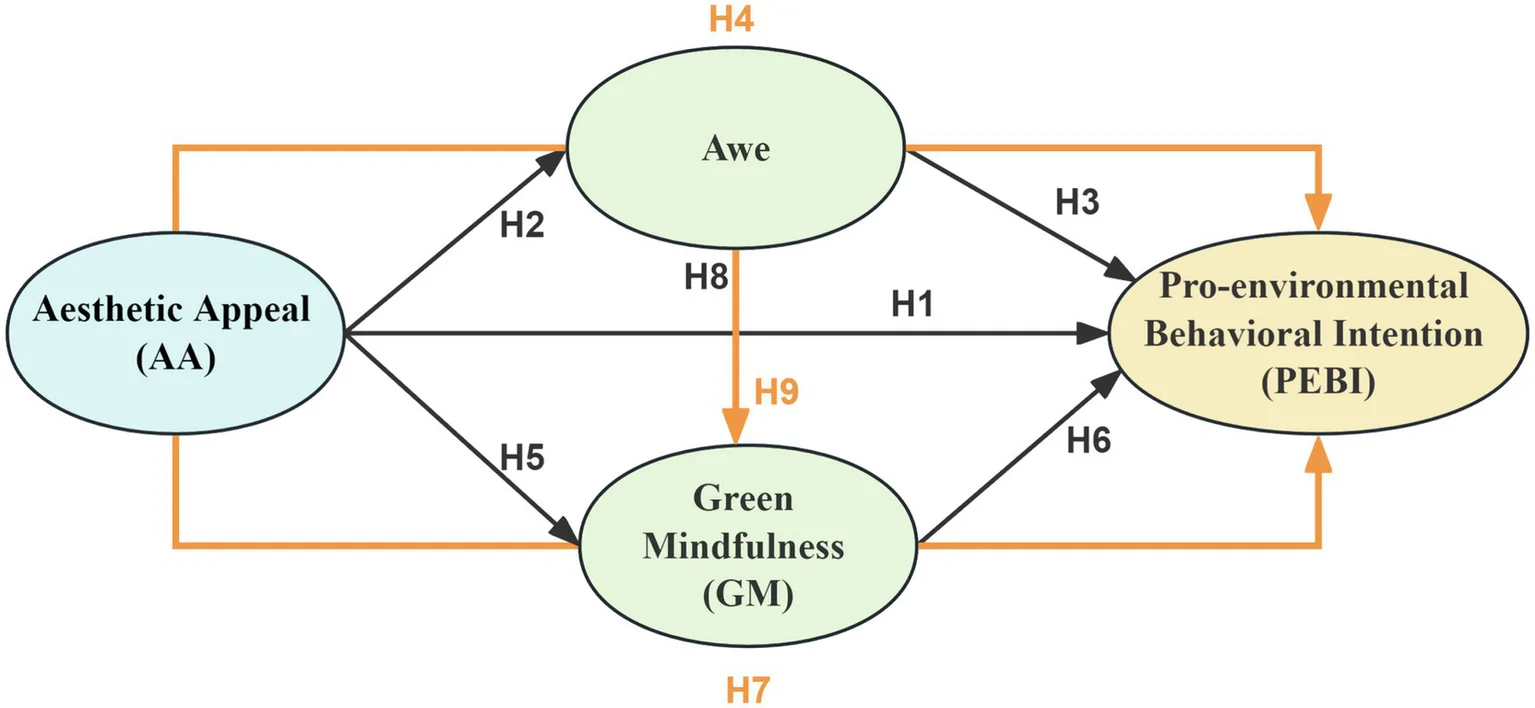
Conceptual model and hypothesized relationships.

## 3 Research methodology and measures

### 3.1 Research context and procedure

The study was embedded in Recitation and Language Arts, a 2-credit general education elective in aesthetic education offered to undergraduate students at Yunnan Agricultural University. The course covers verbal expression, recitation, literary appreciation, and the aesthetics of traditional Chinese poetry. The study focused on the instructional unit devoted to traditional Chinese poetry centered on nature and ecological themes.

Before this unit, students had already been introduced to the cultural background, aesthetic features, and major forms of expression in traditional Chinese poetry through earlier course activities. They also had some experience reading, interpreting, and appreciating poetry. This prior exposure provided the basic knowledge needed to engage with the selected poems and allowed students to report their responses with reference to the learning experience taking place in the course rather than relying mainly on remote prior encounters with poetry.

The study materials consisted mainly of poems by Wang Wei and other classical Chinese poets in whose works nature occupies a central place. The selected poems feature imagery such as landscapes, vegetation, animals, weather, and seasonal change, through which they portray natural settings, seasonal rhythms, and relationships between humans and nature. These features made the poems suitable for examining students’ aesthetic responses to nature-related content and their subsequent environmental perceptions.

During the study session, participants were asked to read the selected poems carefully and to pay particular attention to their natural imagery, aesthetic atmosphere, and cultural meanings. They were given sufficient opportunity to engage with the poems before completing the questionnaire. The questionnaire was then completed on the basis of their experience of reading and appreciating the selected works.

### 3.2 Participants and data collection

Recitation and Language Arts is offered as a university-wide elective course open to undergraduate students from all disciplines. Data were collected over four consecutive academic semesters, in May and November 2024 and May and November 2025. To ensure methodological consistency, the same set of poems, reading duration, instructor, questionnaire, standardized instructions, administration procedure, and data collection protocol were maintained across all four survey periods. The sample therefore included undergraduate students from different academic years and disciplinary backgrounds who participated under consistent instructional and data collection conditions.

The study was conducted in accordance with relevant ethical standards and was approved by the Institutional Ethics Committee (Approval No. 2024-LL-003). Before participation, students were informed about the purpose and procedures of the study and their rights as participants. Participation was voluntary, and students could decline to participate or withdraw at any time without penalty. Written informed consent was obtained from all participants before data collection. Following informed consent, participants received a QR code linking to an online questionnaire administered through the Wenjuanxing platform during the class session. Participants completed the questionnaire independently on their mobile devices following standardized instructions. No personally identifiable information, including names, student identification numbers, or contact details, was collected, and all responses were recorded anonymously and used only for this study and related academic research.

A total of 451 questionnaires were returned. Responses were screened using predefined quality-control criteria, including unusually short completion times, highly repetitive response patterns, and logical inconsistencies across related items. Forty-two questionnaires failed one or more of these criteria and were excluded. The final sample comprised 409 valid responses, which were included in the subsequent analyses.

Participant characteristics are summarized in Table 1. Of the 409 participants, 220 (53.79%) were female and 189 (46.21%) were male. Students from all four undergraduate years were represented, with first-year students constituting the largest subgroup (37.16%). Participants represented 10 academic disciplines, with agricultural sciences, engineering, management, and natural sciences accounting for the largest proportions. Information was also collected on prior exposure to classical Chinese poetry, contact with natural environments, and environmental coursework. Slightly more than half of the participants (52.57%) reported occasional prior exposure to classical Chinese poetry, while 39.61% had completed coursework related to environmental protection, ecology, sustainable development, or related topics. Detailed distributions of these demographic and background characteristics are provided in Table 1.

**Table 1:** Demographic, academic, and prior experience characteristics of participants.

Variable	Category	*n*	%
Gender	Male	189	46.21
	Female	220	53.79
Year of study	First-year undergraduate	152	37.16
	Second-year undergraduate	107	26.16
	Third-year undergraduate	118	28.85
	Fourth-year undergraduate	32	7.83
Academic discipline	Agriculture	107	26.16
	Economics	39	9.54
	Law	18	4.40
	Management	47	11.49
	Education	31	7.58
	Science	44	10.76
	Engineering	68	16.62
	Literature	28	6.85
	Medicine	16	3.91
	Arts	11	2.69
Frequency of exposure to or reading of classical Chinese poetry prior to taking this course.	Almost never	31	7.58
	Rarely	93	22.73
	Occasionally	215	52.57
	Often	62	15.16
	Very often	8	1.96
Frequency of contact with natural environments in daily life (e.g., parks, forests, fields, and mountains).	Almost never	18	4.40
	Rarely	63	15.40
	Sometimes	184	44.99
	Often	96	23.47
	Very often	48	11.74
Previous systematic coursework in environmental protection, ecology, sustainable development, or related subjects.	Yes	162	39.61
	No	247	60.39

The background variables were collected to characterize the sample rather than to serve as explanatory variables in the primary structural model. Because the present study was designed as a theory-driven examination of associations rather than a causal effect model, these variables were not included as additional predictors in the primary structural analyses. Given that the same course context, questionnaire, administration procedure, and data collection protocol were used across all four survey periods, the data were pooled for subsequent analyses.

### 3.3 Measurement instruments

All constructs were measured using multi-item scales adapted from established measures. Because the present study examined participants’ responses in the context of traditional Chinese nature poetry appreciation, item wording was adjusted where necessary to align the referent with this specific context. These adaptations were limited to contextual alignment and were not intended to alter the theoretical meaning of the original constructs. Responses were recorded on a five-point Likert scale ranging from 1 (“strongly disagree”) to 5 (“strongly agree”), with higher scores indicating higher levels of the corresponding construct. The measurement items for all constructs are presented in Table 2.

**Table 2:** Reliability and convergent validity of the measurement model (*N* = 409).

Constructs and items	Factor loading	Cronbach’s α	CR	AVE
Aesthetic Appeal (AA)		0.817	0.891	0.732
AA1: I find this poem aesthetically pleasing.	0.867			
AA2: I like this poem.	0.869			
AA3: I find this poem highly appealing.	0.830			
Awe		0.908	0.932	0.732
Awe1: While reading this poem, I experienced a strong sense of awe.	0.863			
Awe2: The natural world depicted in this poem filled me with wonder.	0.823			
Awe3: While reading this poem, I was struck by the vastness and grandeur of the natural world.	0.862			
Awe4: The natural scenes depicted in this poem made me feel small in comparison.	0.882			
Awe5: The natural world presented in this poem prompted me to reconsider the relationship between humans and nature.	0.846			
Green Mindfulness (GM)		0.879	0.908	0.623
GM1: After reading this poem, I pay greater attention to subtle changes in the natural environment.	0.836			
GM2: After reading this poem, I am more attentive to what is happening in the natural environment around me.	0.801			
GM3: At this moment, I actively pay more attention to information related to the natural environment.	0.800			
GM4: At this moment, I am more willing to consider the relationship between humans and nature from different perspectives.	0.805			
GM5: After reading this poem, I pay greater attention to the potential impact of my behavior on the natural environment.	0.731			
GM6: At this moment, I attend to the natural environment in a more open and conscious manner rather than habitually overlooking it.	0.759			
Pro-Environmental Behavioral Intention (PEBI)		0.846	0.896	0.684
PEBI1: In the future, I am willing to reduce my use of single-use products as much as possible.	0.815			
PEBI2: In the future, I am willing to make a greater effort to conserve resources such as water and electricity.	0.824			
PEBI3: In the future, when circumstances permit, I am willing to prioritize more environmentally friendly products or lifestyles.	0.815			
PEBI4: In the future, I am willing to participate in or support actions aimed at protecting the natural environment.	0.855			

The questionnaire underwent translation and back-translation, followed by expert review and a pilot test. Expert review focused on whether the items were linguistically clear, culturally appropriate, and conceptually consistent with the intended constructs. Particular attention was given to distinguishing aesthetic appeal, state awe, green mindfulness, and pro-environmental behavioral intention (PEBI). The questionnaire was subsequently pilot-tested with 30 university students to assess item clarity and comprehensibility. These students did not participate in the main survey.

Aesthetic appeal (AA) was defined as participants’ subjective appreciation of the aesthetic qualities conveyed by traditional Chinese nature poetry, particularly the perceived beauty, aesthetic attractiveness, and overall liking of nature-related poetic content. Three items were adapted on the basis of measures reported by Schindler et al. (2017) and Wang et al. (2021) to assess perceived beauty, liking, and overall aesthetic attractiveness. In the present study, aesthetic appeal reflected context-specific aesthetic engagement with natural imagery and poetic expression rather than general aesthetic ability or literary knowledge.

State awe was defined as participants’ immediate experience of awe during engagement with the selected poems, particularly in relation to natural imagery and perceptions of vastness and grandeur. Unlike dispositional awe, which reflects an individual’s general tendency to experience awe across situations, state awe captures a context-specific emotional experience occurring during poetry appreciation. Five items were adapted from Shiota et al. (2006), with wording adjusted to refer specifically to participants’ experiences while reading and appreciating traditional Chinese nature poetry rather than to a general disposition to experience awe.

Green mindfulness (GM) was defined as environmentally oriented mindful awareness characterized by attentive awareness of environmental information and consideration of the ecological implications of human actions. Six items were adapted from Chen et al. (2014), with limited contextual modifications to ensure relevance to the nature-related setting of the present study while retaining the original conceptual focus of the construct. Unlike general mindfulness, green mindfulness specifically concerns awareness of environmentally relevant information and the relationship between human actions and environmental consequences.

Pro-environmental behavioral intention (PEBI) was defined as participants’ intentions to engage in environmentally beneficial behaviors. Four items adapted from Yu et al. (2017) assessed intentions related to resource conservation, reduction in waste and disposable-product use, adoption of environmentally friendly lifestyles, and participation in or support for environmental protection activities.

### 3.4 PLS-SEM and ANN hybrid data analysis

The analysis followed a two-stage PLS-SEM–ANN approach combining theory-driven structural assessment with complementary prediction. In the first stage, PLS-SEM was used to assess the measurement model and estimate the hypothesized relationships among the latent constructs. Given the study’s emphasis on examining theoretically specified relationships while retaining a prediction-oriented analytical perspective, PLS-SEM was considered appropriate for the analysis (Hair et al., 2019). All PLS-SEM analyses were conducted using SmartPLS 4.

In the second stage, ANN was employed as a complementary predictive method. Whereas PLS-SEM estimates theoretically specified relationships among latent constructs, ANN can model potentially complex and nonlinear relationships that may not be adequately represented by linear structural specifications. The ANN analysis was therefore used to complement, rather than replace, the theory-driven interpretation provided by PLS-SEM (Morris et al., 2004; Wang et al., 2022). Specifically, ANN was used to evaluate predictive performance and the relative importance of the main predictors of PEBI. It was used exclusively for prediction and predictor-importance analysis and was not used for mediation testing or causal inference. ANN analyses were conducted using SPSS 26.0, following the general SEM–ANN analytical procedures described by Leong et al. (2025).

### 3.5 Common method bias

Because all constructs were measured using the same self-administered questionnaire, potential common method bias (CMB) was addressed through both procedural and statistical remedies. During data collection, procedural measures included ensuring respondent anonymity, informing participants that there were no right or wrong answers, and encouraging them to respond based on their actual experiences.

Harman’s single-factor test was first conducted as a statistical diagnostic of potential CMB. The first unrotated factor accounted for 43.421% of the total variance, which was below the commonly used 50% criterion (Podsakoff et al., 2003). Although Harman’s single-factor test cannot rule out common method bias, this result indicated that no single factor dominated the variance structure. In addition, full-collinearity VIF values ranged from 1.546 to 3.036, remaining below the 3.3 criterion suggested by Kock (2015). Taken together, these diagnostics suggest that common method bias was unlikely to constitute a serious threat to the interpretation of the observed associations.

## 4 Results

### 4.1 Measurement model assessment

Table 2 reports the reliability and convergent validity results for the measurement model. Standardized factor loadings ranged from 0.731 to 0.882, meeting the recommended criteria (Henseler et al., 2016; Sarstedt et al., 2022). Cronbach’s α ranged from 0.817 to 0.908, and composite reliability (CR) ranged from 0.891 to 0.932; both were above the 0.70 criterion. The average variance extracted (AVE) ranged from 0.623 to 0.732, exceeding 0.50 for all constructs. Thus, the four constructs showed adequate internal consistency and convergent validity, supporting the appropriateness of the adapted measures for the specific context of traditional Chinese nature poetry appreciation.

Discriminant validity was examined using the Fornell–Larcker criterion and the heterotrait–monotrait ratio (HTMT). For each construct, the square root of AVE exceeded its correlations with the other constructs (Fornell and Larcker, 1981; Hair et al., 2019). The HTMT values ranged from 0.330 to 0.709, with the highest value (0.709) remaining below the conservative criterion of 0.85 (Sarstedt et al., 2022). Discriminant validity was therefore supported. Together with the satisfactory reliability, validity, and collinearity diagnostics, these results provide support for the robustness of the measurement model (Table 3).

**Table 3:** Fornell–Larcker criterion and HTMT results.

Variable	Fornell–Larcker				HTMT			
	Awe	GM	AA	PEBI	Awe	GM	AA	PEBI
Awe	0.856				–			
GM	0.623	0.789			0.691	–		
AA	0.291	0.500	0.855		0.330	0.587	–	
PEBI	0.411	0.528	0.594	0.827	0.462	0.609	0.709	–

### 4.2 Structural model

The structural model was assessed after establishing the reliability and validity of the measurement model. VIF values ranged from 1.000 to 1.999, showing no indication of problematic multicollinearity. The model explained 8.5% of the variance in awe, 50.0% in green mindfulness, and 43.7% in PEBI (*R*^2^ = 0.085, 0.500, and 0.437, respectively). The corresponding Q^2^ values were 0.061, 0.304, and 0.291. All *Q*^2^ values were positive, supporting the predictive relevance of the model. The standardized root mean square residual (SRMR) was 0.075, below the reference value of 0.10 (Hair et al., 2021).

The significance of the path coefficients in the structural model was tested using bootstrapping with 5,000 resamples. Table 4 and Figure 2 report the direct effects. Aesthetic appeal was positively related to PEBI (*β* = 0.444, *t* = 9.628, *p* < 0.001), supporting H1, and to awe (*β* = 0.291, *t* = 6.766, *p* < 0.001), supporting H2. Awe was also positively associated with PEBI (*β* = 0.148, *t* = 2.459, *p* = 0.014), supporting H3.

**Table 4:** Hypothesis testing results for direct effects.

Hypothesis	Path relationship	*β*	*t*	*p*	CI [2.5–97.5%]	VIF	*f* ^2^	Remarks
H1	AA → PEBI	0.444	9.628	0.000	[0.352, 0.533]	1.335	0.262	Supported
H2	AA → Awe	0.291	6.766	0.000	[0.198, 0.368]	1.000	0.093	Supported
H3	Awe → PEBI	0.148	2.459	0.014	[0.032, 0.270]	1.637	0.024	Supported
H5	AA → GM	0.349	7.939	0.000	[0.259, 0.434]	1.093	0.222	Supported
H6	GM → PEBI	0.214	3.349	0.001	[0.083, 0.334]	1.999	0.041	Supported
H8	Awe → GM	0.522	11.905	0.000	[0.434, 0.605]	1.093	0.498	Supported

**Figure 2:**
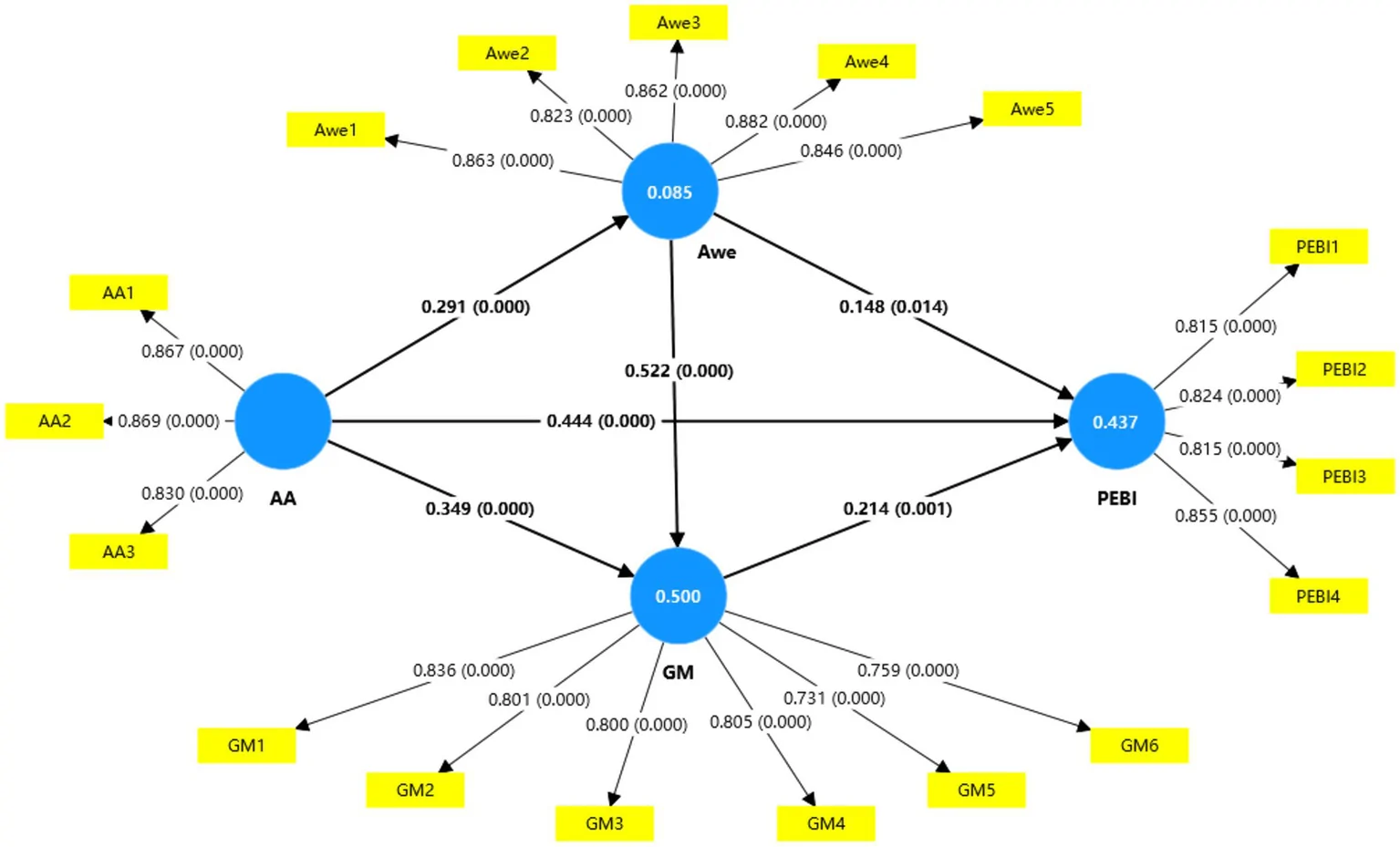
Structural model results.

Aesthetic appeal was positively associated with green mindfulness (*β* = 0.349, *t* = 7.939, *p* < 0.001), supporting H5. Green mindfulness was positively related to PEBI (*β* = 0.214, *t* = 3.349, *p* = 0.001), supporting H6. The strongest direct path in the structural model was from awe to green mindfulness (*β* = 0.522, *t* = 11.905, *p* < 0.001), supporting H8.

### 4.3 Mediation effect test

Indirect effects were assessed using bootstrapping procedures in PLS-SEM following Preacher and Hayes (2008), and the results are reported in Table 5. The indirect association of aesthetic appeal with PEBI through awe was significant (*β* = 0.043, *t* = 2.274, *p* = 0.023, 95% CI [0.009, 0.085]), providing support for H4. The indirect association through green mindfulness was also significant (*β* = 0.075, *t* = 3.106, *p* = 0.002, 95% CI [0.030, 0.124]), providing support for H7. The serial indirect association through AA → Awe → GM → PEBI was significant (*β* = 0.032, *t* = 2.945, *p* = 0.003, 95% CI [0.013, 0.056]), providing support for H9. Because the direct association between aesthetic appeal and PEBI remained significant after the mediators were included in the structural model, these findings indicate partial rather than complete mediation. Therefore, the proposed sequential model accounts for part of the statistical association between aesthetic appeal and PEBI.

**Table 5:** Hypothesis testing results for indirect effects.

Hypothesis	Path relationship	*β*	*t*	*p*	CI [2.5–97.5%]	Remarks
H4	AA → Awe → PEBI	0.043	2.274	0.023	[0.009, 0.085]	Supported
H7	AA → GM → PEBI	0.075	3.106	0.002	[0.030, 0.124]	Supported
H9	AA → Awe → GM → PEBI	0.032	2.945	0.003	[0.013, 0.056]	Supported

### 4.4 Artificial neural network (ANN) analysis

After completing the PLS-SEM analysis, we used an artificial neural network (ANN) to assess the predictive importance of aesthetic appeal, awe, and green mindfulness for pro-environmental behavioral intentions (PEBI) (Leong et al., 2025). The ANN analysis served as a predictive complement to the PLS-SEM results and was used to estimate the relative contributions of these key predictors. Its purpose was predictive rather than causal; it was not intended to provide additional evidence of causal relationships or mediation effects.

The ANN analysis was performed with the Neural Networks module in IBM SPSS Statistics, Version 26. Specifically, a multilayer perceptron (MLP) network was developed using a feedforward architecture and trained with the backpropagation algorithm. The network comprised an input layer, a hidden layer, and an output layer. The sigmoid activation function was used in both the hidden and output layers (Gary, 2024). The input layer contained three neurons representing aesthetic appeal, awe, and green mindfulness, while the output layer contained one neuron for pro-environmental behavioral intentions (PEBI). The model included one hidden layer, with SPSS automatically selecting the number of hidden neurons based on the characteristics of the dataset. This procedure helped reduce the subjectivity associated with manually specifying the hidden-layer structure. Figure 3 presents the architecture of the ANN model.

**Figure 3:**
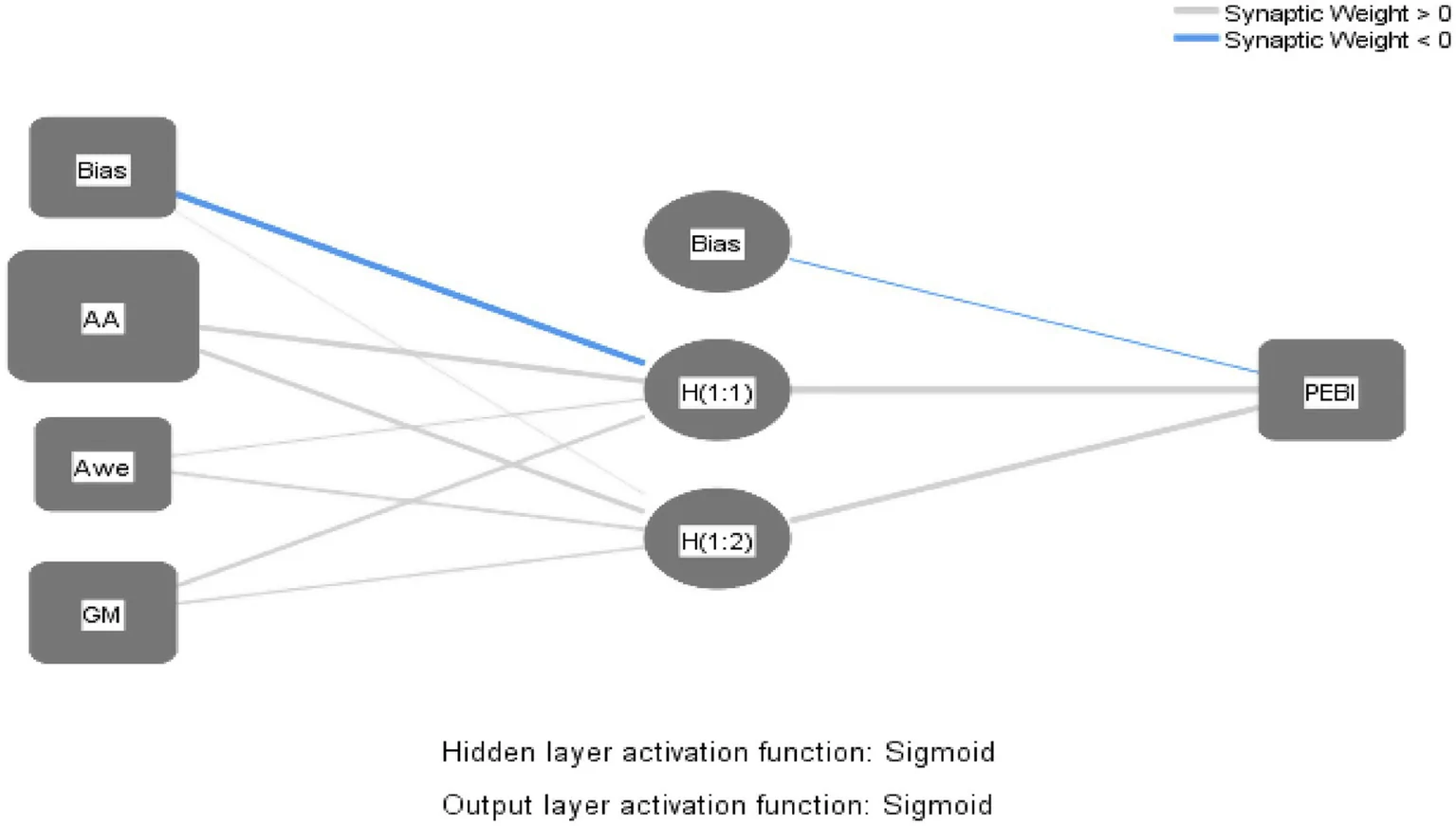
Architecture of the ANN model.

Because ANN results can vary with random data partitioning and may be affected by overfitting, we used 10 repeated training–testing splits to assess the model’s stability and predictive performance. For each run, 90% of the observations were randomly assigned to the training set, and the remaining 10% were allocated to the testing set (Kumar et al., 2023). After each run, the data were repartitioned and the model was retrained. This procedure generated 10 sets of training and testing results based on different random partitions of the same dataset. During training, the algorithm iteratively updated the network weights through gradient descent to minimize prediction errors.

Table 6 shows that the RMSE values ranged from 0.113 to 0.126 across the training sets and from 0.094 to 0.146 across the testing sets. The mean RMSE was 0.117 (SD = 0.004) for the training sets and 0.118 (SD = 0.016) for the testing sets. Although the testing-set errors were more variable, the similar mean RMSE values demonstrate that no overfitting occurred and suggest that the model maintained comparable predictive performance on unseen observations.

**Table 6:** Prediction performance of ANN models based on RMSE.

Neural network	Input: AA, Awe, GM; Output: PEBI					
	Training			Testing		
	*N*	SSE	RMSE	*N*	SSE	RMSE
ANN1	365	4.672	0.113	44	0.654	0.122
ANN2	367	5.321	0.12	42	0.375	0.094
ANN3	367	5.181	0.119	42	0.456	0.104
ANN4	358	4.781	0.116	51	0.668	0.114
ANN5	357	4.869	0.117	52	0.505	0.099
ANN6	370	5.92	0.126	39	0.679	0.132
ANN7	364	4.638	0.113	45	0.616	0.117
ANN8	368	5.366	0.121	41	0.868	0.146
ANN9	362	4.853	0.116	47	0.712	0.123
ANN10	370	4.693	0.113	39	0.661	0.13
Mean		5.029	0.117		0.619	0.118
SD		0.412	0.004		0.141	0.016

N, sample size; SSE, sum of squares of errors; RMSE, root mean square of errors; SD, standard deviation.

Table 7 presents the sensitivity analysis, while Table 8 compares the ANN and PLS-SEM results. Aesthetic appeal recorded the highest normalized relative predictive importance (100.00%), followed by green mindfulness (51.93%) and awe (31.41%). These scores describe each predictor’s relative contribution to ANN prediction and should not be interpreted as evidence of causal effects. The PLS-SEM results showed the same ranking, with aesthetic appeal having the largest path coefficient, followed by green mindfulness and awe. Because the ANN importance scores and PLS-SEM path coefficients capture different analytical properties, their numerical magnitudes should not be compared directly. Nevertheless, both methods yielded a consistent ranking of the three predictors.

**Table 7:** ANN sensitivity analysis based on relative predictive importance.

Neural network	AA	Awe	GM
ANN1	0.527	0.206	0.267
ANN2	0.559	0.129	0.312
ANN3	0.517	0.156	0.327
ANN4	0.614	0.157	0.229
ANN5	0.552	0.2	0.248
ANN6	0.365	0.252	0.383
ANN7	0.539	0.159	0.301
ANN8	0.634	0.163	0.203
ANN9	0.628	0.112	0.26
ANN10	0.519	0.179	0.302
Average importance	0.545	0.171	0.283
Normalized relative importance (%)	100.00%	31.41%	51.93%

**Table 8:** Comparison of PLS-SEM and ANN results.

PLS-SEM path	PLS-SEM: path coefficient	ANN: normalized relative importance (%)	Ranking (PLS-SEM)	Ranking (ANN)	Remark
AA → PEBI	0.444	100%	1	1	Matched
Awe → PEBI	0.148	31.41%	3	3	Matched
GM → PEBI	0.214	51.93%	2	2	Matched

## 5 Discussion

This study examined the associations among perceived aesthetic appeal, awe, green mindfulness, and pro-environmental behavioral intention (PEBI) within the context of traditional Chinese nature poetry appreciation. Three main findings emerged. First, aesthetic appeal was positively associated with PEBI, awe, and green mindfulness. Second, both awe and green mindfulness showed significant indirect associations between aesthetic appeal and PEBI. Third, a significant serial indirect association involving aesthetic appeal, awe, green mindfulness, and PEBI was identified. The direct association between aesthetic appeal and PEBI remained significant after accounting for the specified indirect pathways. The ANN analysis provided complementary predictive evidence, identifying aesthetic appeal as the predictor with the greatest relative importance, followed by green mindfulness and awe. Together, these findings highlight theoretically meaningful relationships among aesthetic evaluation, emotional experience, environmentally oriented awareness, and behavioral intention within a culturally embedded educational context.

### 5.1 The indirect association involving awe

The significant indirect association involving awe is consistent with previous research linking nature-related awe to environmentally relevant responses (Yang et al., 2018; Chirico et al., 2023). Traditional Chinese nature poetry frequently presents mountains, rivers, the sky, the moon, and seasonal changes through imagery conveying vastness and transcendence. Such representations provide a meaningful cultural context in which aesthetic appreciation and awe may be related. Students who evaluate these poems more positively may also report stronger emotional engagement with their natural imagery and poetic atmosphere. Given that perceived vastness is a central characteristic of awe-related experiences (Keltner and Haidt, 2003), this connection offers a plausible interpretation of the positive association between aesthetic appeal and awe observed in the present study.

Awe was also positively associated with PEBI. One possible explanation can be drawn from the “small-self” perspective, which suggests that awe is associated with reduced self-focus and greater attention to concerns extending beyond immediate personal interests (Piff et al., 2015). This perspective is relevant to environmental intentions because environmentally responsible choices often involve consideration of broader ecological and collective consequences. Previous research has linked awe to ecological behavior through connectedness to nature (Yang et al., 2018), while Monroy and Keltner (2023) highlighted broader psychological relationships involving awe, self-transcendence, and connectedness.

Within the present study, students reporting stronger awe during poetry appreciation also tended to express stronger pro-environmental intentions. The significant indirect association suggests that awe may help account statistically for part of the relationship between perceived aesthetic appeal and PEBI. Although this finding is consistent with an affective interpretation of nature-related aesthetic appreciation, it does not establish that aesthetic evaluation produced awe or that awe subsequently caused stronger behavioral intentions.

### 5.2 The indirect association involving green mindfulness

Green mindfulness also showed a significant indirect association between perceived aesthetic appeal and PEBI. This finding suggests that environmentally oriented awareness may be relevant to understanding the relationship between aesthetic evaluation and environmental intentions. Aesthetic appreciation involves perceptual and cognitive evaluation rather than judgments of beauty alone (Leder et al., 2004). In traditional Chinese nature poetry, landscapes, vegetation, animals, and seasonal changes constitute important elements of poetic imagery. Students who find such poetry aesthetically appealing may also report greater attentiveness to nature-related information and human–nature relationships. This interpretation is compatible with the positive association between aesthetic appeal and green mindfulness observed in the present study.

Green mindfulness was positively associated with PEBI, consistent with previous research connecting mindfulness and environmentally oriented awareness with pro-environmental intentions and behaviors (Apaolaza et al., 2022; Bernadip et al., 2026). Green mindfulness emphasizes conscious attention to environmental information and reflection on the ecological implications of human actions. Individuals reporting greater environmentally oriented awareness may therefore also express stronger intentions to conserve resources, reduce waste, and make environmentally responsible choices.

The significant indirect association involving green mindfulness provides statistical support for examining environmental awareness alongside aesthetic evaluation and behavioral intention. However, the observed relationship does not establish that appreciating poetry increases green mindfulness. Rather, it indicates that students reporting more positive aesthetic evaluations also tended to report greater environmentally oriented awareness, which was positively associated with PEBI.

### 5.3 The serial indirect association involving awe and green mindfulness

The significant serial indirect association is noteworthy because it suggests that awe and green mindfulness may be meaningfully related within the broader association between aesthetic appeal and PEBI. Specifically, aesthetic appeal was positively associated with awe, awe was positively associated with green mindfulness, and green mindfulness was positively associated with PEBI. These findings are consistent with a theoretically specified model incorporating both emotional experience and environmentally oriented awareness.

Awe and green mindfulness represent distinct but potentially complementary psychological constructs. Awe is primarily characterized by self-transcendent emotional experience, whereas green mindfulness involves conscious awareness of environmental information and human–nature relationships (Chen et al., 2014; Piff et al., 2015). The positive association between awe and green mindfulness suggests that students reporting stronger self-transcendent emotional experiences also tended to report greater environmental attentiveness.

One possible theoretical interpretation can be drawn from the broaden-and-build perspective, which suggests that positive emotional experiences can broaden attention and expand the range of thoughts and actions available to individuals (Fredrickson, 2001). Although awe should not be equated with positive emotion in general, this perspective provides a conceptual basis for considering possible relationships between emotional experience and attentional orientation. Within traditional Chinese nature poetry appreciation, natural imagery and representations of human–nature relationships may provide meaningful content relevant to both awe and green mindfulness.

Importantly, the significant serial indirect association should be understood as a statistical pattern consistent with the hypothesized relationships rather than evidence of a confirmed psychological sequence. The findings support further investigation of how self-transcendent emotion and environmentally oriented awareness may be related in aesthetic and sustainability education.

### 5.4 The association between aesthetic appeal and pro-environmental behavioral intention

Perceived aesthetic appeal remained positively associated with PEBI after awe and green mindfulness were included in the structural model. This finding is broadly consistent with previous research linking appreciation of natural beauty to environmentally relevant intentions and behaviors (Diessner et al., 2018; Diessner and Niemiec, 2023). However, appreciation of natural environments and aesthetic evaluation of poetic representations are not identical constructs, and this distinction should be considered when interpreting the findings.

Traditional Chinese nature poetry integrates natural imagery with cultural meanings and emotional expression. Mountains, rivers, vegetation, and seasonal changes contribute to the aesthetic character of these poems and may provide opportunities for reflection on relationships between humans and nature. Students who report greater appreciation of such poetic representations may also express stronger environmentally responsible intentions. Nevertheless, perceived aesthetic appeal was measured as an evaluation of the poetry’s beauty, attractiveness, and overall appeal, rather than as a direct measure of environmental values or ecological understanding.

The remaining direct association indicates that the statistical relationship between aesthetic appeal and PEBI was not fully accounted for by the specified indirect pathways. Other psychological and contextual variables, including connectedness to nature, environmental values, cultural identification, and perceived ecological meaning, may be relevant to this relationship (Xie, 2023). These possibilities warrant further investigation.

The ANN findings provided complementary predictive evidence. Aesthetic appeal showed the greatest normalized relative predictive importance, followed by green mindfulness and awe, consistent with their ordering in the PLS-SEM results. Nevertheless, ANN importance values and structural path coefficients represent different analytical properties and should not be interpreted as equivalent measures of explanatory or causal influence. The convergence in predictor ordering therefore strengthens the descriptive characterization of the observed predictive relationships without establishing causal priority among the constructs.

### 5.5 Practical implications

The findings offer several potential implications for integrating traditional Chinese nature poetry into aesthetic and sustainability education. These implications concern possible educational applications rather than demonstrated effects of poetry-based interventions.

First, nature-oriented poetry could provide culturally meaningful materials for connecting traditional cultural education with environmental topics. Educators may encourage students to explore how natural imagery, poetic atmosphere, and representations of human–nature relationships contribute to the aesthetic meanings of classical poetry. Such activities could provide opportunities for discussing contemporary environmental concerns while maintaining the cultural and literary significance of the works.

Second, poetry appreciation could be combined with complementary educational activities involving direct engagement with natural environments. Recitation, imagery-based interpretation, nature observation, and campus ecological activities represent possible approaches for connecting aesthetic appreciation with environmental reflection. Rather than treating awe as an outcome that can be reliably produced through poetry exposure, educators could provide opportunities for students to consider experiences of wonder, vastness, and human–nature relationships.

Third, the positive association between green mindfulness and PEBI highlights the relevance of environmentally oriented awareness in sustainability education. Reflective discussions, resource-conservation activities, and consideration of everyday environmental choices may offer opportunities for students to examine the ecological implications of their actions. Such activities could complement traditional approaches emphasizing environmental knowledge by incorporating attention to environmental information and personal responsibility.

Nevertheless, the effectiveness of these educational approaches remains to be established. Future intervention studies should examine whether poetry-based educational activities influence environmental awareness, behavioral intentions, and actual environmentally responsible behavior.

### 5.6 Limitations and future directions

Several limitations should be considered when interpreting the findings.

First, the study did not independently evaluate the effects of traditional Chinese nature poetry exposure. All participants engaged with the same selected poetry materials under standardized instructional conditions, without an experimental manipulation or a comparison or control condition. Although participants reported different levels of perceived aesthetic appeal, these differences cannot be attributed specifically to the poetry experience. They may also reflect pre-existing individual characteristics, including aesthetic preferences, previous familiarity with poetry, and environmental orientations. Accordingly, traditional Chinese nature poetry should be understood as the shared cultural and educational context of the study rather than an empirically tested stimulus. Future experimental studies could compare nature-related poetry with non-nature poetry, alternative cultural materials, or appropriate control conditions to examine whether specific poetic experiences influence aesthetic evaluations and environmentally relevant psychological outcomes.

Second, although data were collected across four academic semesters, each participant completed the questionnaire only once. The cross-sectional design therefore prevents the establishment of temporal ordering or causal relationships among aesthetic appeal, awe, green mindfulness, and PEBI. In particular, statistically significant indirect associations do not constitute evidence of causal mediation. The hypothesized ordering represents one theoretically informed specification, while alternative directions and explanations remain possible. Longitudinal studies with repeated measurements and experimental designs incorporating suitable manipulations would provide stronger evidence for evaluating the proposed psychological relationships.

Third, the sample was drawn from undergraduate students enrolled in a general education course at Yunnan Agricultural University in Yunnan Province, China. The findings may therefore reflect characteristics of this specific educational and cultural setting and should not be assumed to generalize to other student populations. Additionally, although the same instructional and data collection procedures were maintained across four academic semesters, cohort-related variation cannot be completely excluded. Future research should examine whether similar relationships are observed across different universities, academic disciplines, geographic regions, and cultural contexts.

Fourth, the study assessed pro-environmental behavioral intention rather than directly observed environmental behavior. Although behavioral intention is commonly considered an important antecedent of behavior, actual environmentally responsible actions may also depend on available resources, situational constraints, and competing priorities. Future research could incorporate behavioral observations, records of environmental choices, or follow-up assessments to evaluate whether the observed associations extend to actual behavior.

Fifth, all constructs were assessed using a single self-administered questionnaire. Although procedural precautions and statistical diagnostics were employed to assess potential common method bias, these approaches cannot eliminate all sources of shared-method variance or self-report bias. Furthermore, some green mindfulness items referred to participants’ perceived attentiveness after reading the poems, but no corresponding pre-exposure measurements were collected. Such responses therefore cannot establish actual changes in mindfulness attributable to poetry appreciation. Future studies could incorporate baseline assessments, repeated measurements, behavioral indicators, and data obtained from additional sources.

Sixth, the conceptual model focused on awe and green mindfulness, while other potentially relevant psychological and contextual variables were not examined. Previous research suggests that place attachment may be relevant to relationships between eco-emotions and pro-environmental behavior (Bontempo et al., 2026b). Future research could investigate place attachment alongside connectedness to nature, environmental values, cultural identification, ecological responsibility, and perceived ecological meaning to develop a more comprehensive understanding of the observed associations.

Finally, several demographic and experiential characteristics were collected but were not included as explanatory variables in the primary structural model. These included prior poetry exposure, frequency of contact with natural environments, previous environmental coursework, academic discipline, and year of study. Such characteristics may be associated with aesthetic evaluations, emotional experiences, environmental awareness, and behavioral intentions. Future studies could examine their potential explanatory or moderating roles and compare different forms of cultural and aesthetic experience to clarify the generalizability and contextual specificity of the observed relationships.

## 6 Conclusion

This study examined the associations among perceived aesthetic appeal, awe, green mindfulness, and pro-environmental behavioral intention (PEBI) in the context of traditional Chinese nature poetry appreciation. Perceived aesthetic appeal was positively associated with PEBI, while awe and green mindfulness showed significant indirect associations, both independently and sequentially. These findings underscore the relevance of self-transcendent emotion and environmentally oriented awareness in linking aesthetic evaluation with pro-environmental intention.

By integrating aesthetic appeal, awe, and green mindfulness within a culturally embedded educational context, this study offers a more nuanced understanding of the psychological correlates of PEBI. However, the observed indirect effects should be interpreted as statistical associations rather than evidence of causal mediation. The cross-sectional design also precludes conclusions about the causal effects of poetry exposure or the temporal ordering of the proposed processes. Future experimental and longitudinal research is therefore needed to determine whether poetry-based educational experiences can influence environmental awareness, behavioral intentions, and actual pro-environmental behavior.

## Data Availability

The raw data supporting the conclusions of this article will be made available by the authors, without undue reservation.
